# LncRNA LINC-PINT Inhibits Malignant Behaviors of Laryngeal Squamous Cell Carcinoma Cells via Inhibiting ZEB1

**DOI:** 10.3389/pore.2021.584466

**Published:** 2021-04-01

**Authors:** Xianguang Yang, Susheng Miao, Xionghui Mao, Cheng Xiu, Ji Sun, Rong Pei, Shenshan Jia

**Affiliations:** Department of Head and Neck Surgery, Harbin Medical University Cancer Hospital, Harbin, China

**Keywords:** laryngeal squamous cell carcinoma, ZEB1, migration, invasion, EZH2, LINC-PINT

## Abstract

**Objective:** Laryngeal squamous cell carcinoma (LSCC) belongs to head and neck squamous cell carcinoma (HNSCC), with dismal prognosis. Here, this study aims to disclose the role of LINC-PINT in cancer development, which may contribute to improving the clinical outcomes of LSCC treatment.

**Methods:** LINC-PINT expression in LSCC tissues and in TU-177 and Hep-2 cells was quantified, and subsequently, the association between LINC-PINT and LSCC malignancies was analyzed. pcDNA3.1-LINC-PINT or pcDNA3.1-EZH2 was introduced into Hep-2 and TU-177 cells. qRT-PCR and Western blot analyses examined the levels of proteins related to the AKT/mTOR pathway and their phosphorylated proteins in Hep-2 and TU-177 cells. The viability as well as migration and invasion abilities of Hep-2 and TU-177 cells were determined. Also, the distribution of LINC-PINT in Hep-2 cells was investigated as well as the interplay between LINC-PINT and EZH2. The downstream genes that might interact with EZH2 were screened.

**Results:** LINC-PINT expression was inhibited in LSCC tissues and in Hep-2 and TU-177 cells, whose downregulation was associated with unsatisfactory prognosis. LINC-PINT overexpression suppressed the proliferative, migratory and invasive capacities of Hep-2 and TU-177 cells. LINC-PINT, mainly expressing in nuclei, could enrich EZH2 to silence ZEB1. In Hep-2 and TU-177 cells, the inhibition of LINC-PINT or overexpression of ZEB1 could enhance cell proliferation, migration and invasion. The phosphorylated levels of proteins related to the AKT/mTOR pathway were declined in cells with LINC-PINT overexpression, and the levels of these phosphorylated proteins were increased in cells with LINC-PINT inhibition.

**Conclusion:** LINC-PINT enriches EZH2 to silence ZEB1 and thus inhibits the proliferative, migratory, and invasive capacities of Hep-2 and TU-177 cells. In addition, LINC-PINT might exert its biological function through the AKT/mTOR pathway.

## Introduction

Laryngeal squamous cell carcinoma (LSCC) is a common subtype of head and neck squamous cell carcinoma (HNSCC), with increased incidence year by year [1, 2]. The firstly considered therapeutic strategy for laryngeal carcinoma is nonsurgical approaches since loss of larynx brings functional morbidity [3, 4]. However, the recurrence rate for patients with advanced LSCC is still high even after primary radiotherapy and chemotherapy [5]. Hence, a potent therapeutic target for LSCC treatment is urgently required to improve the long-term therapeutic and functional outcomes for LSCC patients.

Long noncoding RNAs (lncRNAs) with over 200 nucleotides in length have been widely concerned for their versatile functions on the development of tumors [6]. To our knowledge, multiple lncRNAs have been demonstrated to promote LSCC through enhancing the biological processes of LSCC cells, including cell proliferation, migration and invasion [7–9]. Intriguingly, LINC-PINT, which is transcriptionally regulated by p53 has been found to restrain cancer cell stemness and chemoresistance to cisplatin in laryngeal carcinoma [10]. Moreover, in esophageal squamous cell carcinoma (ESCC), the recurrence of ESCC is connected to downregulated LINC-PINT expression, whose upregulation suppresses the migration and invasiveness of ESCC cells [11]. However, it requires more efforts to explore the functional role and probable action mechanism of LINC-PINT in LSCC.

Methyltransferase enhancer of zeste homolog 2 (EZH2) is a well-known catalyzer for polycomb repressive complex 2 (PRC2) that could modulate the oncogenic effect of XIST in LSCC [8]. In recent years, the pivotal role of EZH2 in various cancers has been noticed due to its vital function on mediating the histone methyltransferase activity of PRC2 [12–14]. In melanoma cell lines, EZH2 is the only functional protein that binds to LINC-PINT in the nuclei, and LINC-PINT exerts an inhibitory effect on the pathogenesis of melanoma through enriching EZH2 to the promoter of its target genes [15]. Moreover, an evidence suggested that EZH2 mediates tumor progressions through regulating the expression levels of multiple genes, including KLF2, DKK-1, CDKN1C, ZEB1, ACE2, Robo4, P4HA1, and DUXAP8 [16–23].

Based on the aforementioned evidences, we hypothesized that EZH2 might bind the promoter of its downstream genes and regulate the expression levels of KLF2, DKK-1, CDKN1C, ZEB1, ACE2, Robo4, P4HA1, and DUXAP8 to mediate LSCC development. The current study investigated the impact of LINC-PINT on EZH2 expression. We also explored whether LINC-PINT could recruit EZH2 to the promoter of the aforementioned genes and then regulated the expression levels of these genes in the LSCC cell line TU-177 and Hep-2 carcinoma cells. This study was adopted to understand the roles and possible mechanisms of LINC-PINT in cancer development with regard to the regulation of ZEB1 expression.

## Materials and Methods

### Human Tissue Specimens

LSCC tissues and corresponding adjacent normal margin (ANM) tissues were from patients (*n* = 30) who underwent partial or total laryngectomy in the Department of Head and Neck Surgery of Harbin Medical University Cancer Hospital from August 2016 to May 2017. Patients included in this study had neither radiotherapy/chemotherapy history before operation nor clinical treatment before being diagnosed. The tissues were preserved in liquid nitrogen within 5 min after excision and maintained at −80°C for later use. The ethics committee of Harbin Medical University Cancer Hospital approved the experiments in this study, and written consent was acquired from each patient. This study was conducted in obedience to the Declaration of Helsinki, and a follow-up study was performed for 3 years.

### Cell Culture and Transfection

An LSCC cell line TU-177 and a Hela derivative Hep-2 (from American Type Culture Collection, ATCC) and normal human oral keratinocytes (NHOKs, from Shanghai Institute of Biochemistry and Cell Biology, Chinese Academy of Sciences) were cultured in RPMI1640 (72400120, GIBCO, NY, United States) containing 10% fetal bovine serum (FBS, 16140071, GIBCO, NY, United States) at 37°C in a 5% CO_2_ incubator.

pcDNA3.1 plasmid with LNC-PINT overexpression (pcDNA3.1-LINC-PINT), ZEB1 overexpression (pcDNA3.1-ZEB1) and EZH2 overexpression (pcDNA3.1-EZH2), short-hairpin RNA of LINC-PINT (sh-LINC-PINT) and EZH2 (sh-EZH2) and corresponding negative controls (pcDNA3.1-NC and sh-NC) were bought from Shanghai Genechem Co., Ltd. (Shanghai, China). Cells were cultured in 60 mm culture dishes for 24 h at a density of 3.0 × 10^5^ cells/dish. Afterward, the cells were cultured with 3 μg of the aforementioned plasmid or shRNA, Lipofectamine 2,000 reagent (11668019, Invitrogen, CA, United States), Opti-MEM I Reduced Serum Medium (31985062, GIBCO, NY, United States) and 8 ng/ml polybrene (TR-1003, Sigma-Aldrich, St. Louis, MO, United States) for 48 h.

The cells were accordingly assigned into Control group, pcDNA3.1-LINC-PINT group, sh-LINC-PINT group, pcDNA3.1-EZH2 group, sh-EZH2 group, pcDNA3.1-ZEB1 group and pcDNA3.1-LINC-PINT + pcDNA3.1-ZEB1 group.

### CCK-8

Proliferation rate of cells were measured by a CCK-8 kit (Beyotime, Shanghai, China). Transfected cells (3,000 cells/well) were pipetted into a 96-well plate, and fresh culture medium was replaced every day. Then, the proliferation rate of the cells were detected by the CCK-8 kit every 24 h for 5 consecutive days. In short, 10 μL of CCK-8 solution was added into each well and cultured with the cells for 2 h at 37°C, after which the absorbance was measured at 450 nm using the microplate reader SpectraMax M5 (Molecular Devices).

### qRT-PCR

Tissues or cells were lyzed in 1 ml of Trizol reagent (Thermo Fisher Scientific, MA, United States) and total RNAs were isolated following the manufacturer’s directions. Reverse transcription was conducted to obtain cDNA with the assistance of M-MLV reverse transcriptase and arbitrary primers. Reaction conditions for qRT-PCR were configured in line with the instructions of Premix Ex Taq™II kit (Takara, Dalian, China), and the ABI7500 quantitative PCR instrument (Applied Biosystems, Shanghai, China) was employed for RT-PCR analysis. GAPDH was used for normalization, and relative mRNA levels of genes were analyzed by 2^−ΔΔCt^ method. ΔΔCt = [Ct _(target gene)_–Ct _(internal reference)_]_experimental group_–[Ct _(target gene)_–Ct _(internal reference)_]_control group_. Names and sequences of primers used in this study were listed (Table 1).

**TABLE 1:** Names and sequences of all primers.

Name of primer	Sequences (5′-3′)
LINC-PINT-F	CGT​GGG​AGC​CCC​TTT​AAG​TT
LINC-PINT-R	GGG​AGG​TGG​CGT​AGT​TTC​TC
EZH2-F	AGT​CAC​TGG​TCA​CCG​AAC​AC
EZH2-R	TTG​GGT​AGG​CAG​CAT​CTC​TT
ZEB1-F	TCG​GAA​AGA​GCT​GTT​CGC​TT
ZEB1-R	AGG​AGG​GGG​CTG​ACA​TAC​AT
CDKN1C-F	CAC​CTT​GGG​ACC​AGT​GTA​CC
CDKN1C-R	CTC​CTC​GCA​GTT​TAG​AGC​CC
KLF2-F	CGG​GAG​GAG​AGG​TCG​GAT​T
KLF2-R	AGA​CTG​TCT​CCC​TAG​CCA​CG
DKK-1-F	TCC​TAC​TGT​CTT​CTC​CTT​CGT
DKK-1-R	GCA​CAA​CAC​AAT​CCT​GAG​GC
ACE2-F	GTG​GTG​GTG​GTA​TCG​GAG​TG
ACE2-R	ACA​GCA​GTA​GCC​TGT​ACT​TCG
Robo4-F	ATC​ACC​AGC​AAC​ACC​CCA​AA
Robo4-R	GGT​TTT​CTG​GCA​AAC​TCG​CA
P4HA1-F	CCA​AGC​CAC​AGG​TGA​TTG​GA
P4HA1-R	TGG​CTG​TTC​TTA​CTG​CCA​CTT
DUXAP8-F	TTA​GTC​TGA​TGC​CGT​GGG​TG
DUXAP8-R	GCT​TCC​TTA​GTG​AGC​TTT​CCC
GAPDH-F	GTG​GCT​GGC​TCA​GAA​AAA​GG
GAPDH-R	GGG​GAG​ATT​CAG​TGT​GGT​GG

F, forward; R, reverse.

### Western Blotting

Tissues and cells were lyzed to obtain proteins using lysis buffer, and the protein concentration was then quantified by a BCA kit (23,227, Thermo Fisher, United States). Subsequently, the proteins were diluted by 5 × loading buffer and separated in 12% separating gel using electrophoresis for 90 min. After being blocked by 1 × PBS containing 5% (w/v) skim milk powder at room temperature for 1 h, the proteins were incubated with primary antibodies, including anti-EZH2 antibody (1:500, ab186006, Cambridge, United Kingdom), anti-ZEB1 antibody (1:500, ab203829, Cambridge, United Kingdom), anti-AKT antibody (1:500, ab38449, abcam, Cambridge, United Kingdom), anti-p-AKT antibody (1:500, ab8805, abcam, Cambridge, United Kingdom), anti-mTOR antibody (1:500, ab32028, abcam, Cambridge, United Kingdom), anti-p-mTOR antibody (1:500, ab109268, abcam, Cambridge, United Kingdom), anti-RPS6KB1 antibody (S6K1, 1:500, ab32529, abcam, Cambridge, United Kingdom), and anti-p-RPS6KB1 antibody (p-S6K1, 1:500, ab59208, abcam, Cambridge, United Kingdom) at 4°C overnight. Thereafter, the proteins were rinsed and incubated with the secondary antibody (1:500, ab150077, abcam, Cambridge, United Kingdom) at room temperature for 1 h before being photographed on BioSpectrum Imaging System (UVP, United States).

### Purification of Cytoplasmic and Nuclear RNA

A Cytoplasmic and Nuclear RNA Purification Kit (cat no.21000; Norgen Biotek, Thorold, ON, United States) was used for the purification of nuclear and cytoplasmic RNA as previously described [24]. Purification was based on spin column chromatography. In brief, after the culture medium was removed, cells were washed with PBS once and lyzed by 200 μL of ice-cold Lysis Buffer J on ice for 5 min. The lysate was then pipetted into a microcentrifuge tube and centrifuged at 14,000 rpm and 4°C for 10 min. The supernatant containing cytoplasmic RNA was transferred into another RNase-free microcentrifuge tube, and the pellet containing nuclear RNA was retained. The tube containing cytoplasmic RNA or nuclear RNA was added 200 μL or 400 μL of Buffer SK, respectively, and then the cytoplasmic or nuclear RNAs were mixed with Buffer SK by vortexing for 10 s. The mixture was then mixed with 200 μL of 98% ethanol by vortexing for 10 s. Thereafter, the mixture was applied onto a spin column and centrifuged at 1,000 rpm for 1 min, after which the flowthrough was discarded. Wash Solution A (400 μL) was added to the column for 1 min of centrifugation and then the flowthrough was discarded, which was repeated twice to wash column. Afterward, the column was centrifuged for 2 min and then dried. The column was placed into an Elution tube and 50 μL of Elution Buffer E was added to the column. The tube was centrifuged at 2,000 rpm for 2 min, followed by 1 min of centrifugation at 14,000 rpm. Finally, the purified RNA was collected and quantified by qRT-PCR.

### Scratch Assay

Cells (1 × 10^5^) from the experimental and control groups were pipetted into 12-well plates and a scratch was made by a 10 μL pipette tip vertically when the cell confluence reached 100%. Exfoliated cells and cell debris were washed off by DPBS (14190250, GIBCO, NY, United States) three times, and then the adherent cells were cultured in fresh DMEM supplemented with 2% FBS. Then, the cells were observed by an Olympus inverted microscope at 0 and 24 h under the same field to assess the changes of the scratch. Migration rate = (gap between the scratch at 0 h–gap between the scratch at 24 h)/gap between the scratch at 0 h. The results were from three independent experiments.

### Transwell Assay

Invasiveness and migratory capacity of Hep2 and TU-177 cells were measured by using a Transwell (Corning, NY, United States). After transfection, cells (3 × 104) in the experimental and control groups were suspended in culture medium containing 1% FBS and seeded onto an apical chamber. Then, 0.8 ml of culture medium with 10% FBS was added onto a basolateral chamber. The insert used for Transwell invasion assay was encased with matrigel at 37°C for 2 h. Twenty four hours later, cells on the surface of the apical membrane were wiped off, and the cells passing the insert were maintained in 4% paraformaldehyde for 30 min. After being fixed, the cells were dyed with 10% Giemsa and rinsed with PBS three times and observed using an inverted microscope. The number of invasive cells was counted at the magnification of × 200 from 5 random fields and then averaged. The number of cells invaded and migrated in the control group was defined as 1, and normalized fold changes of invasive and migratory cells in the experimental groups were accordingly calculated. The results were acquired from three independent experiments.

### RNA Binding Protein Immunoprecipitation (RIP)

A RIP kit was purchased from Guangzhou Saicheng Biotechnology Co., Ltd. (KT102–01, Guangdong, China). Approximate 4 × 10^7^ cells were homogenized with lysis buffer (1 ml) and rotated in a 4°C refrigerator for 1–2 h. Afterward, the cells were centrifuged at 4°C and 14,000 rpm for 10 min, and 10 μL of the supernatant was collected and termed Input group. A total volume of 100 μL of the supernatant was incubated with bead-antibody (5 μg, ab191250, ab75974, ab108252, abcam, Cambridge, and United Kingdom) complexes at 4°C overnight, and then the supernatant was removed on a magnetic rack. Subsequently, 1 ml of RIP buffer was added to wash the complexes and then the supernatant was discarded. Thereafter, each group (including Input group) was incubated with 117 μL of RIP buffer, 15 μL of 10% SDS, and 18 μL of proteinase K at 65°C for 45 min before being centrifuged at 3,000 rpm for 5 min. The supernatant from IP group were added with 250 μL of RIP wash buffer and 400 μL of the homogenate of phenol, chloroform and isoamylol (125:24:1) and mixed by vortexing. After being maintained at room temperature for 5 min, the mixture was centrifuged at 12,000 rpm and room temperature for 15 min. Upper aqueous phase (400 μL) was collected and then mixed with 1.2 ml of absolute ethanol and 2 μL of glycogen for precipitation overnight at −20°C. After precipitation, the mixture was centrifuged at 12,000 rpm and 4°C for 30 min. The supernatant was discarded before the beads were rinsed with 75% ethanol. The beads were centrifuged again, and the supernatant was removed. Finally, the beads were dissolved in DEPC, and RNAs were analyzed by qRT-PCR.

### RNA-Fluorescence *in situ* Hybridization

Specific FISH was conducted using LINC-PINT probes. The 5′CY3 labeled LINC-PINT probes were synthesized by Shanghai Genechem Co., Ltd. (Shanghai, China). All operations were made according to the instruction of the FISH kit (F03401, Genechem, Shanghai, China). Briefly, cells on the slides were fixed with ice-cold 4% paraformaldehyde and degraded at 70°C. The cells were incubated with 30 μg/ml LINC-PINT probes and then counterstained with DAPI after the residual probes were washed off. Finally, the cells were visualized and photographed under a fluorescent microscope.

### Co-Immunoprecipitation Assay

Cells were lyzed to extract proteins, and then the isolated proteins were incubated with anti-EZH2 antibody (ab186006, abcam, Cambridge, United Kingdom) and 20 μL of protein-A/G-agarose beads at 4°C overnight. Following incubation, the precipitation was washed with lysis buffer four times and suspended in 5 × SDS-PAGE loading buffer. The suspension was boiled for 5 min before Western blot analysis.

### Statistical Analysis

Statistical analysis was carried out by using SPSS 18.0 (IBM Corp., Armonk, NY, United States) and GraphPad Prism 6.0 (GraphPad Software Inc.). Results in this study were from three independent experiments unless otherwise stated and the data were presented as average ± standard deviation. *T*-test measured the differences between two groups, and comparisons among multiple groups were assessed using One-way analysis of variance. *P* < 0.05 had statistical significance.

## Results

### Downregulated mRNA LINC-PINT Level Associates With LSCC Development

**FIGURE 1:**
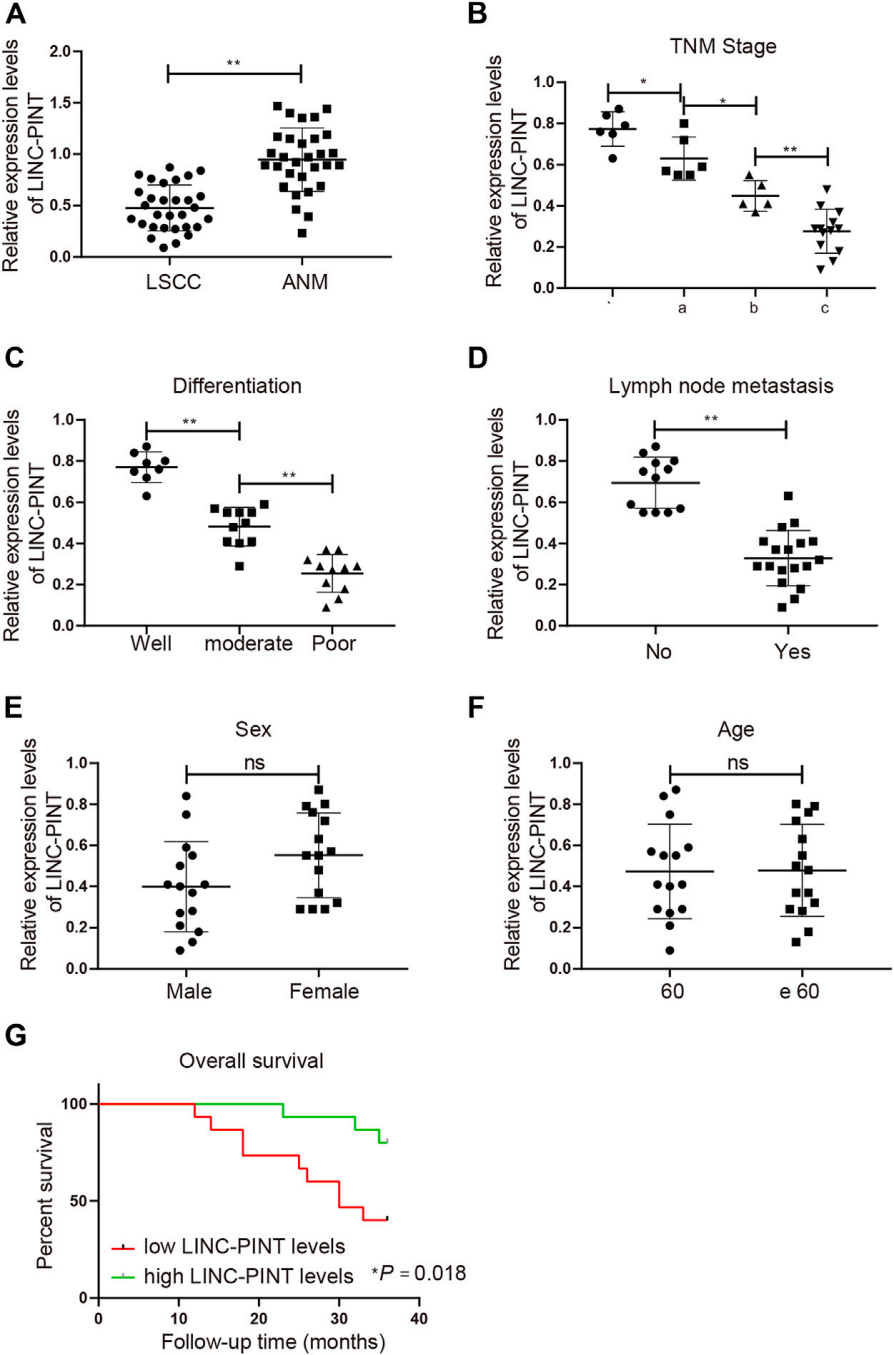
Downregulation of LINC-PINT associates with the poor prognosis of LSCC patients. Note: qRT-PCR analysis of LINC-PINT expression in LSCC and ANM tissues (**A**); the association between LINC-PINT expression and the clinicopathologic features of the LSCC patients, including TNM stage (**B**), differentiation ability (**C**) and lymph mode metastasis (**D**); the association between LINC-PINT expression and sex (**E**) or age (**F**) of the LSCC patients; LINC-PINT expression had a certain relationship with the prognosis of LSCC patients (**G**). *P < 0.05, **P < 0.01. LSCC, laryngeal squamous cell carcinoma; ANM, adjacent normal margin; TNM, tumor node metastasis.

The clinical data of 30 patients enrolled in this study are presented in Table 2. To disclose the clinical significance of LINC-PINT in LSCC, we measured the relative mRNA level of LINC-PINT in LSCC tissues (*n* = 30) and in the corresponding ANM tissues (*n* = 30). The result of qRT-PCR analysis suggested that the mRNA level of LINC-PINT was dramatically downregulated in the LSCC tissues compared with the ANM tissues (Figure 1A, *p* < 0.01), which was consistent with the previous study [10]. To validate the association between LINC-PINT expression and LSCC malignancies, the clinicopathologic features of patients, including tumor node metastasis (TNM) stage, differentiation ability and lymph mode metastasis, were analyzed. With the median LINC-PINT expression as the cutoff value, patients were stratified into the high LINC-PINT group (*n* = 15) and low LINC-PINT group (*n* = 15). As presented in Figures 1B–D, the patients with lower LINC-PINT expression had advanced TNM stage and poor ability of differentiation (*P* < 0.01). The incidence of lymph node metastasis was much higher in patients with lower LINC-PINT expression when compared with those with higher LINC-PINT expression (*P* < 0.01). While there was no significant association between the expression of LINC-PINT and clinical variables such as age and sex (Figures 1E,F, *P* > 0.05). Moreover, the prognostic analysis based on the follow up data indicated that the lower expression of LINC-PINT predicted poor prognosis of LSCC patients (Figure 1G, *P* < 0.01). All these manifested that downregulated LINC-PINT expression was associated with LSCC progression.

**TABLE 2:** Clinical data of 30 patients enrolled in this study.

		Number (n)
Age (y)	<60	15
	≥60	15
Sex	Male	15
	Female	15
TNM stage	Ⅰ	6
	Ⅱ	6
	Ⅲ	5
	Ⅳ	13
Differentiation	Well	8
	Moderate	11
	Poor	11
Lymph node metastasis	Yes	18
	No	12

TNM, tumor node metastasis.

### LINC-PINT Perturbs the Proliferative, Migratory and Invasive Capacities *in vitro*

**FIGURE 2:**
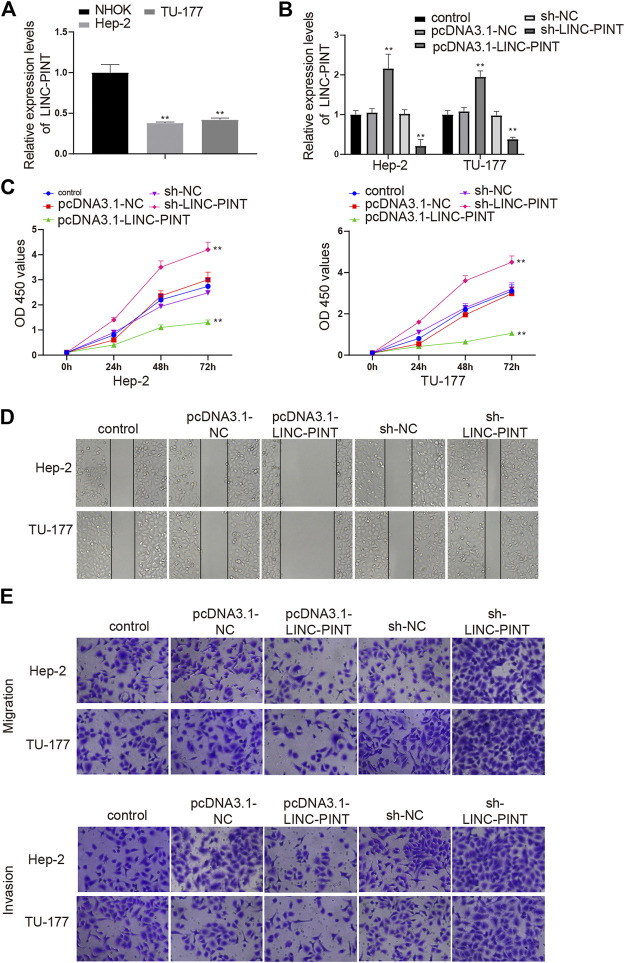
LINC-PINT mediates cell proliferation, migration and invasion. Note: Hep-2 and TU-177 cells were transfected with pcDNA3.1-LINC-PINT and sh-LINC-PINT. qRT-PCR detected the expression of LINC-PINT in Hep-2 and TU-177 cells (**A**) and measured the transfection efficiencies of the pcDNA3.1-LINC-PINT and sh-LINC-PINT (**B**); CCK-8 assay was performed to measure the cell viability of Hep-2, TU-177, and NHOK cells (**C**); the invasion and migration abilities of Hep-2 and TU-177 cells were determined by Scratch assay and Transwell assay (**D–E**). Scale bar = 50 μm **P < 0.01 vs. the Control group. NHOK, normal human oral keratinocytes.

To determine the functional role of LINC-PINT in cancer development, a Hela derivative Hep-2 and an LSCC cell line TU-177 were selected. qRT-PCR analysis of LINC-PINT expression exhibited a great decrease in Hep-2 and TU-177 cells compared to that in NHOK cells (Figure 2A, *P* < 0.01), which was consistent with the detection in LSCC tissues. We next overexpressed or inhibited LINC-PINT in Hep-2 and TU-177 cells. qRT-PCR suggested satisfactory transfection efficiencies of the pcDNA3.1-LINC-PINT and sh-LINC-PINT, as evidenced by increased level of LINC-PINT in the pcDNA3.1-LINC-PINT group and declined expression of LINC-PINT in the sh-LINC-PINT group (Figure 2B, *P* < 0.01). Then, we evaluated the viability of Hep-2 and TU-177 cells by CCK-8 assay. The results indicated that LINC-PINT overexpression suppressed the viability of Hep-2 and TU-177 cells, and LINC-PINT inhibition enhanced the cell viability (Figure 2C, *P* < 0.01). We also detected the influence of LINC-PINT on the migration and invasion of Hep-2 and TU-177 cells through performing Scratch and Transwell assays. Cells transfected with sh-LINC-PINT seemed to be more invasive than those in the Control group and the migration ability were also increased in the sh-LINC-PINT group; overexpression of LINC-PINT appeared to suppress the migration ability and invasiveness of Hep-2 and TU-177 cells (Figures 2D,E; Supplementary Figures S1A,B, *P* < 0.01). Overall, these data supported that overexpression of LINC-PINT suppressed cancer development *in vitro*.

### LINC-PINT Inhibits EZH2 Which Regulates ZEB1 Expression

**FIGURE 3:**
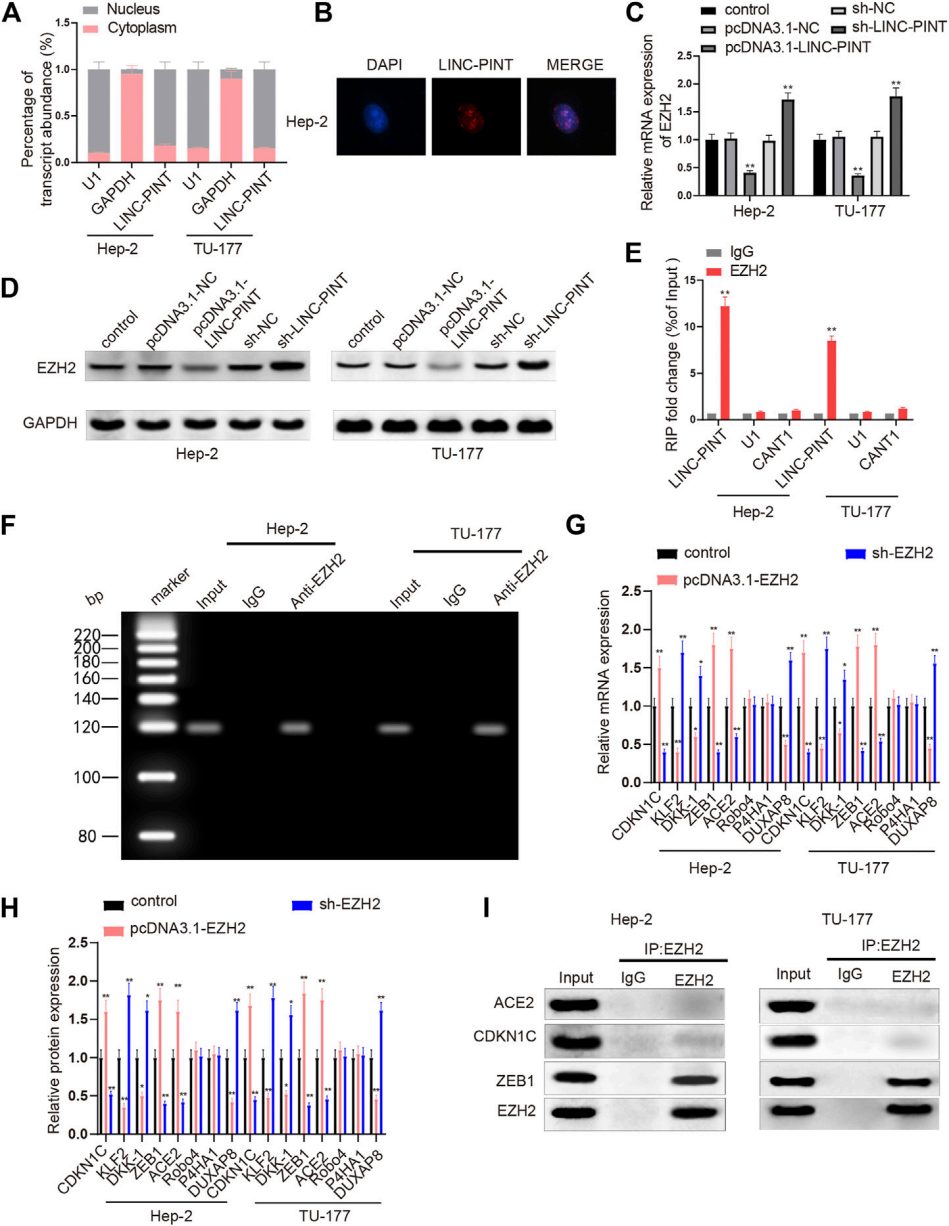
LINC-PINT enriches EZH2 to inhibit ZEB1. Note: Analysis of nuclear/cytoplasmic distribution of LINC-PINT and qRT-PCR analysis verified that LINC-PINT mainly expressed in the nuclei of Hep-2 and TU-177 cells (**A**); RNA-FISH examined the distribution of LINC-PINT in Hep-2 cells (**B**); the impact of LINC-PINT on EZH2 expression in Hep-2 and TU-177 cells was determined by qRT-PCR and Western blotting (**C–D**); the interaction between LINC-PINT and EZH2 in both Hep-2 and TU-177 cells was verified by RIP assay (**E**); PCR analysis quantified gene expression in total RNAs from the RIP product of Hep-2 and TU-177 cells (**F**); qRT-PCR and Western blotting was employed to screen the downstream genes of EZH2 in Hep-2 and TU-177 cells (**G–H**); Co-IP assay was used to determine the proteins interacting with EZH2 in Hep-2 and TU-177 cells (**I**). Scale bar = 50 μm **P < 0.01. Co-IP, Co-immunoprecipitation.

To further illuminate the underlying mechanism by which LINC-PINT regulates cancer development, we investigated the target genes of LINC-PINT. Initially, the content of LINC-PINT in the cytoplasm and nuclei of Hep2 and TU-177 cells was analyzed by qRT-PCR analysis. The results revealed that LINC-PINT mainly expressed in the nuclei of Hep-2 and TU-177 (Figure 3A, *P* < 0.01), which was further identified by the results of RNA-FISH in Hep2 cells (Figure 3B). To investigate the role of EZH2 in LINC-PINT regulating cancer development, we measured the impact of LINC-PINT on EZH2 expression. qRT-PCR and Western blotting found that EZH2 was lowly expressed in the pcDNA3.1-LINC-PINT group and highly expressed in the sh-LINC-PINT group, compared to the Control group (Figures 3C,D; Supplementary Figure S2, *P* < 0.05). In addition, anti-EZH2 antibody was incubated with the total RNAs from Hep-2 and TU-177 cells to conduct RIP assay, and then qRT-PCR analysis was applied to quantify LINC-PINT expression. The results denoted that incubation of anti-EZH2 antibody increased the expression of LINC-PINT in Hep-2 and TU-177 cells compared to the cells incubated with anti-IgG antibody (Figures 3E,F, *P* < 0.01), which confirmed that LINC-PINT specifically bound EZH2.

Next, we screened the downstream genes of EZH2 to explore which genes were regulated by EZH2 in the LSCC cell line TU-177 and Hep-2 carcinoma cells. Hep-2 and TU-177 cells were cultured with pcDNA3.1-EZH2 or sh-EZH2, and then the expression levels of CDKN1C, ACE2, Robo4, KLF2, P4HA1, DUXAP8, DKK-1, and ZEB1 were quantified by qRT-PCR and Western blotting. In cells transfected with pcDNA3.1-EZH2, the expression levels of CDKN1C, ACE2, and ZEB1 were increased, while the levels of DUXAP8, DKK-1, and KLF2 were downregulated; Hep2 and TU-177 cells from the sh-EZH2 group presented massive decreases in the levels of CDKN1C, ACE2, and ZEB1 as well as increases in the levels of DUXAP8, DKK-1, and KLF2 (Figures 3G,H, *P* < 0.01); no significant changes were noticed in the expression levels of Robo4 and P4HA1 either in the pcDNA3.1-EZH2 or in the sh-EZH2 groups. CDKN1C, ACE2, and ZEB1 were selected for Co-IP assay to address the possible interaction with EZH2. Anti-EZH2 antibody was incubated with the proteins extracted from Hep-2 and TU-177 cells, and then we quantified the expression levels of CDKN1C, ACE2, and ZEB1 in the protein-antibody complexes. As exhibited in Figure 3I, the relative levels of CDKN1C and ACE2 were hardly detected while ZEB1 was abundant in the protein-antibody complexes. Taken together, LINC-PINT suppressed EZH2 expression, and the enhancer of EZH2 bound the promoter of ZEB1 to regulate ZEB1 expression.

### LINC-PINT Suppresses Cancer Cell Proliferative, Migratory, and Invasive Capacities Through ZEB1

**FIGURE 4:**
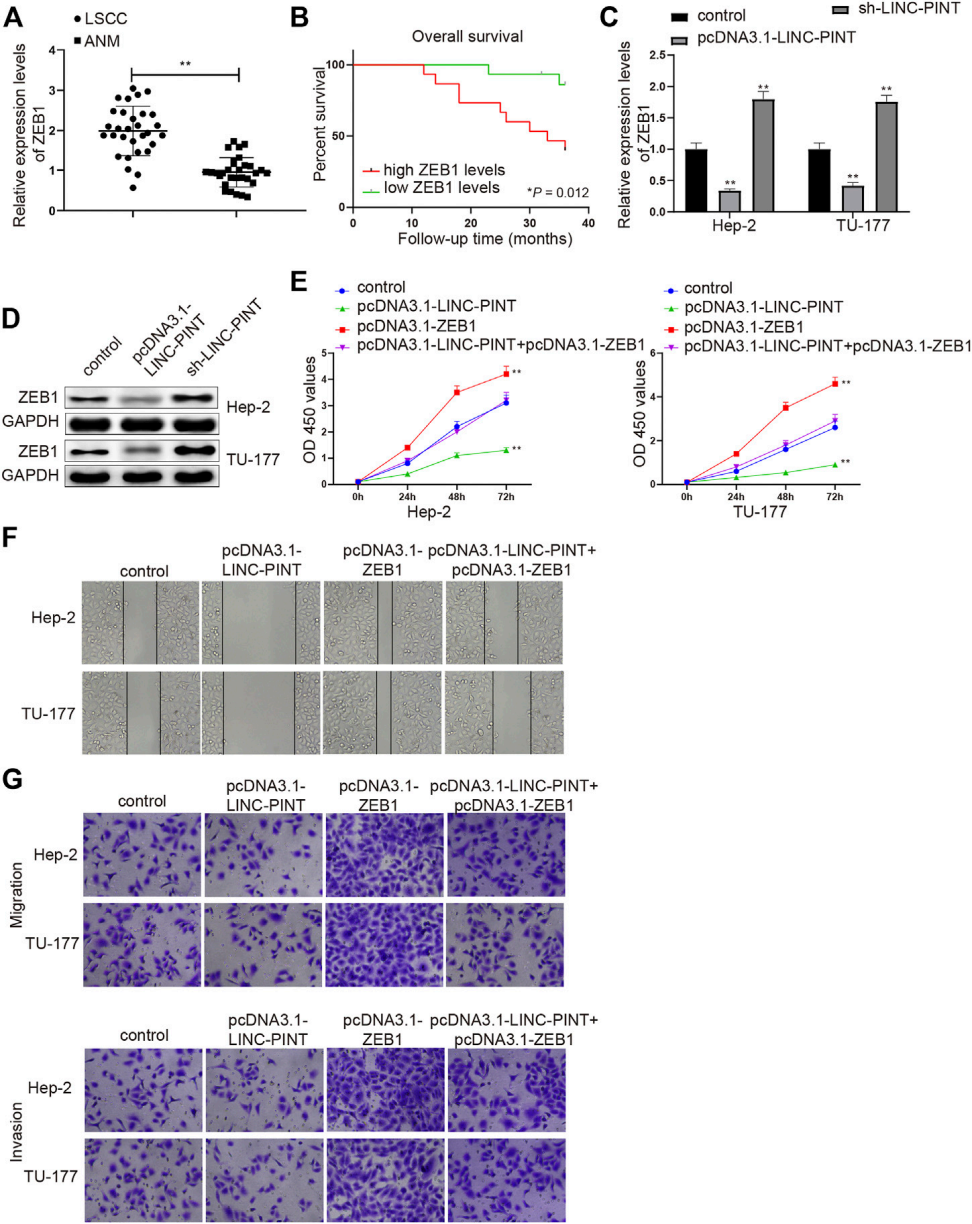
ZEB1 silencing inhibits cell migration and invasion. Note: qRT-PCR analysis detected the mRNA expression of ZEB1 in LSCC and ANM tissues (**A**); the association between ZEB1 expression and the prognosis of LSCC patients (**B**); qRT-PCR and Western blotting quantified the mRNA (**C**) and protein (**D**) expression levels of ZEB1 in Hep-2 and TU-177 cells transfected with pcDNA3.1-LINC-PINT or sh-LINC-PINT (**C–D**); After Hep-2 and TU-177 cells were transfected with pcDNA3.1-ZEB1, pcDNA3.1-LINC-PINT and pcDNA3.1-LINC-PINT + pcDNA3.1-ZEB1, CCK-8, Scratch, and Transwell assays measured the cell viability (**E**), migration (**F**), and invasion (**G**) abilities, respectively. Scale bar = 50 μm **P < 0.01 vs. the Control group. LSCC, laryngeal squamous cell carcinoma; ANM, adjacent normal margin.

The expression of ZEB1 in LSCC tissues was measured to disclose the effect of ZEB1 on LSCC progression. Compared with the ANM tissues, the mRNA level of ZEB1 was upregulated in the LSCC tissues (Figure 4A, *P* < 0.01). The patients were divided into high ZEB1 group (*n* = 15) and low ZEB1 group (*n* = 15), with the median ZEB1 expression as the cutoff value. The prognostic analysis manifested that higher expression of ZEB1 implied dismal prognosis of LSCC patients (Figure 4B, *P* < 0.01).

Based on the aforementioned findings, we next revealed whether LINC-PINT could inhibit cancer cell propagation, invasion and migration via ZEB1. Hep-2 and TU-177 cells were, respectively, introduced with pcDNA-3.1-LINC-PINT and sh-LINC-PINT, and then the expression of ZEB1 was assessed by qRT-PCR and Western blot analyses. Gain- or loss-of function experiment manifested that the mRNA and protein expression levels of ZEB1 were downregulated in the pcDNA3.1-LINC-PINT group and upregulated in the sh-LINC-PINT group (Figures 4C,D; Supplementary Figure S3A, *P* < 0.01), suggesting that LINC-PINT expression was inversely associated with ZEB1 in Hep2 and TU-177 cells.

Then, Hep-2 and TU-177 cells were transfected with pcDNA3.1-LINC-PINT, pcDNA3.1-ZEB1 or co-transfected with pcDNA3.1-LINC-PINT and pcDNA3.1-ZEB1, and cell proliferation, migration and invasion abilities were detected. As expected, the propagation, and invasion and migration rates of Hep2 and TU-177 cells were enhanced in the pcDNA3.1-ZEB1 group (vs. the Control group), and the cell viability and invasion and migration abilities were also increased in the pcDNA3.1-LINC-PINT + pcDNA3.1-ZEB1 group compared to the pcDNA3.1-LINC-PINT group (Figures 4E–G; Supplementary Figures S3B,C, *P* < 0.01). These data supported that the upregulation of ZEB1 could counteract, in part, the inhibitory action of LINC-PINT on the malignant properties of Hep2 and TU-177 cells.

### LINC-PINT Blocks the AKT/mTOR Pathway *in vitro*

**FIGURE 5:**
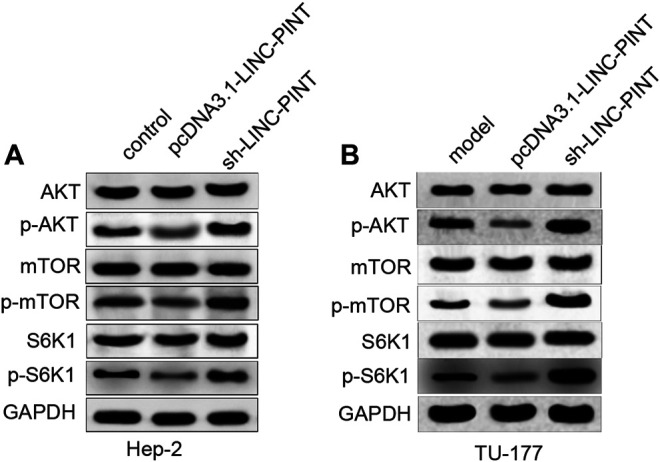
LINC-PINT regulates the AKT/mTOR signaling *in vitro*. Note: After Hep-2 and TU-177 cells were transfected with pcDNA3.1-LINC-PINT and sh-LINC-PINT, the expression levels of proteins related to the AKT/mTOR signaling pathway were quantified by Western blotting **(A–B)**. **P < 0.01 vs. the Control group.

To elucidate the mechanism regarding LINC-PINT regulating cancer progression, we determined the expression levels of AKT, mTOR, RPS6KB1 (S6K1) and their phosphorylated proteins in Hep-2 and TU-177 cells transfected with pcDNA3.1-LINC-PINT and sh-LINC-PINT. As shown in Figure 5A; Supplementary Figures S4A,B, the phosphorylated levels of AKT, mTOR and S6K1 were downregulated in the pcDNA3.1-LINC-PINT group, and significant increases in the phosphorylated levels of AKT, mTOR, and S6K1 were found in the sh-LINC-PINT group, compared to the Control group (*P* < 0.01).

## Discussion

Admittedly, ectopic expression of lncRNAs is responsible for various malignant processes in LSCC [25, 26]. In recent years, much attention has been attracted to utilize these molecules as therapeutic targets. The tumor suppressive role of LINC-PINT has been illuminated in cancers [27, 28], whereas, the knowledge for its role in LSCC is still insufficient. Herein, LINC-PINT was illustrated as a suppressor of LSCC and exerted its role through inhibiting ZEB1. In addition, the AKT/mTOR signaling pathway was also unearthed to be activated after the downregulation of LINC-PINT.

Firstly, LINC-PINT expression was identified to be inhibited both in LSCC tissues and Hep-2 and TU-177 cells. The downregulation of LINC-PINT related to dismal prognosis and unfavourable clinicopathological characteristics, including advanced TNM stage, differentiation and lymph node metastasis for LSCC patients. Our *in vitro* experiments exhibited that upregulated LINC-PINT expression perturbed the proliferation, migration and invasion of Hep-2 and TU-177 cells. The significant role of LINC-PINT in cancers has been well illustrated. For example, the decreased expression of LINC-PINT has been noticed in esophageal cancer, leading to advanced tumor and node stage and miserable overall survival [29]. LINC-PINT overexpression also suppresses the biological performance of non-small cell lung cancer cells such as cell proliferation, migration and invasion, which has been corroborated by *in vivo* experiments [30]. In LSCC, we observed that LINC-PINT was downregulated in LSCC patients and cells, and its expression was negatively associated with LSCC progression.

We next observed that LINC-PINT could bind and downregulate EZH2. EZH2 is an enzymatic subunit of PRC2, and PRC2 is a complex that promotes transcriptional silencing through methylating lysine 27 of histone H3 [31]. P52-regulated lncRNA LINC-PINT has a highly conserved sequence that is significant for its tumor suppressive role through specifically interacting with PRC2 [32]. The interaction between LINC-PINT and EZH2 has been mentioned in clear cell renal cell carcinoma (ccRCC). LINC-PINT is referred to directly bind EZH2 and the knockdown of EZH2 partially counteracts the promoting effects of LINC-PINT overexpression on the malignant behaviors of ccRCC cells [33]. The oncogenic properties of LINC-PINT in ccRCC suggested that LINC-PINT might have different roles in different disease background. LncRNA HOTAIR recruits EZH2 to specific target genes and induces the trimethylation of H3K27 and epigenetic silencing of genes that suppress breast cancer metastasis [34]. Our experiments proved that EZH2 could enrich ZEB1 in Hep-2 and TU-177 cells. Ectopic ZEB1 is sufficient for the silencing of interferon regulatory factor 1 (IRF1) through the catalytic activity of EZH2, which impacts chronic oxidative injury and triggers epithelial-mesenchymal transition (EMT) after the occurrence of airway diseases [35]. ZEB1 is an important transcription factor in regulating EMT, and it has been speculated to cooperate with OIP5-AS1 and induce EMT of LSCC cells [36]. More importantly, the overexpression of ZEB1 has been found in lung squamous cell cancer, whose aberrant expression is involved in the occurrence, development and invasion of lung squamous cell cancer [37]. The overexpression of LINC-PINT inhibited ZEB1 expression. The upregulation of ZEB1 expression was revealed to partly reverse the suppressive effects of LINC-PINT overexpression on cancer cell proliferation, invasion and migration abilities. Collectively, this study confirmed that LINC-PINT recruited EZH2 to the promoter of ZEB1, by which LINC-PINT downregulated ZEB1 and inhibited the malignant behaviors of Hep-2 and TU-177 cells.

In addition, LINC-PINT inhibition increased the phosphorylated levels of the AKT/mTOR pathway-related proteins. The activation of the AKT/mTOR signaling pathway is thought to enhance cancer cell proliferation in LSCC [38]. Overexpression of EZH2 is able to activate the PI3K/AKT pathway, by which BRCA1 (a tumor suppressor) were exported from nuclei, and aneuploidy and mitotic deficiency were elicited, leading to invasive breast cancer [39]. Liu et al has evidenced that increased expression of ZEB1 could significantly abolish miR-199a-5p-inhibited EMT of ovarian endometriotic stromal cells through activating the PI3K/AKT/mTOR signaling pathway [40]. In future study, we would verify the impact of LINC-PINT/EZH2/ZEB1 on the activation of the AKT/mTOR signaling pathway.

In summary, the present study quantified the changes of LINC-PINT expression in LSCC tissues and in the LSCC cell line TU-177 and Hep-2 carcinoma cells and elucidated the inhibitory functions and action mechanism of LINC-PINT on LSCC development, suggesting a prognostic marker and a potent therapeutic direction for LSCC treatment.

## Data Availability

The authors acknowledge that the data presented in this study must be deposited and made publicly available in an acceptable repository, prior to publication. Frontiers cannot accept a article that does not adhere to our open data policies.
