# Supplementary figures and images for: LncRNA LINC-PINT Inhibits Malignant Behaviors of Laryngeal Squamous Cell Carcinoma Cells via Inhibiting ZEB1
Source: Pathol Oncol Res. 2021 Apr 1;27:584466. doi: 10.3389/pore.2021.584466 (PMC8262191; doi:10.3389/pore.2021.584466)

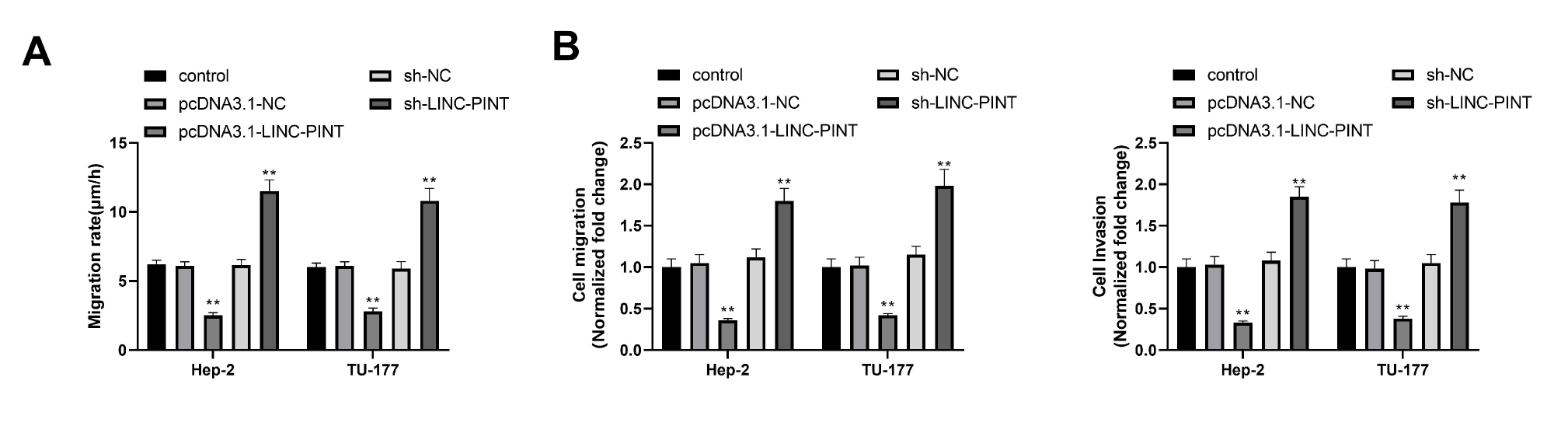

Supplement: Supplementary file 1 [file Image1.TIF]

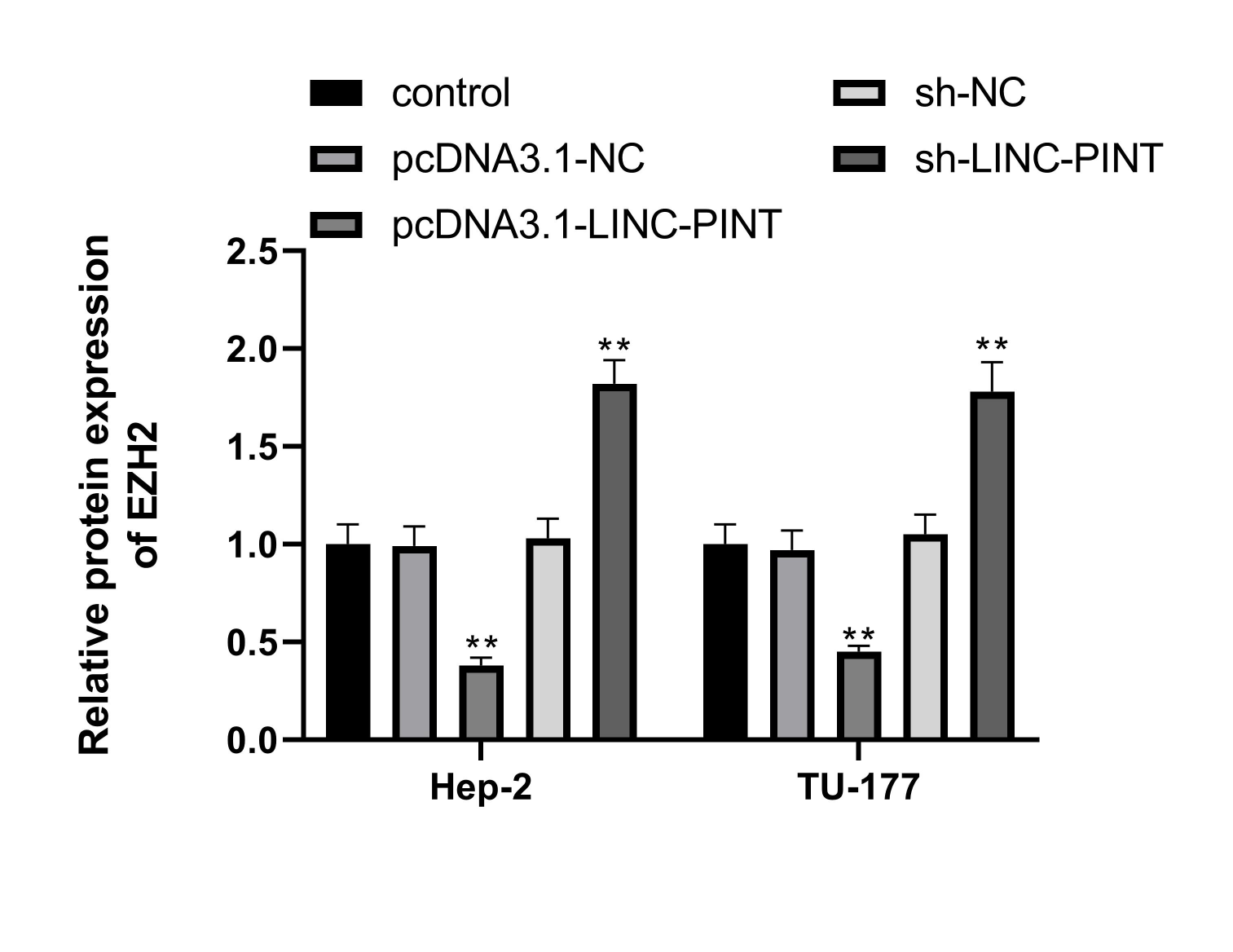

Supplement: Supplementary file 2 [file Image2.TIF]

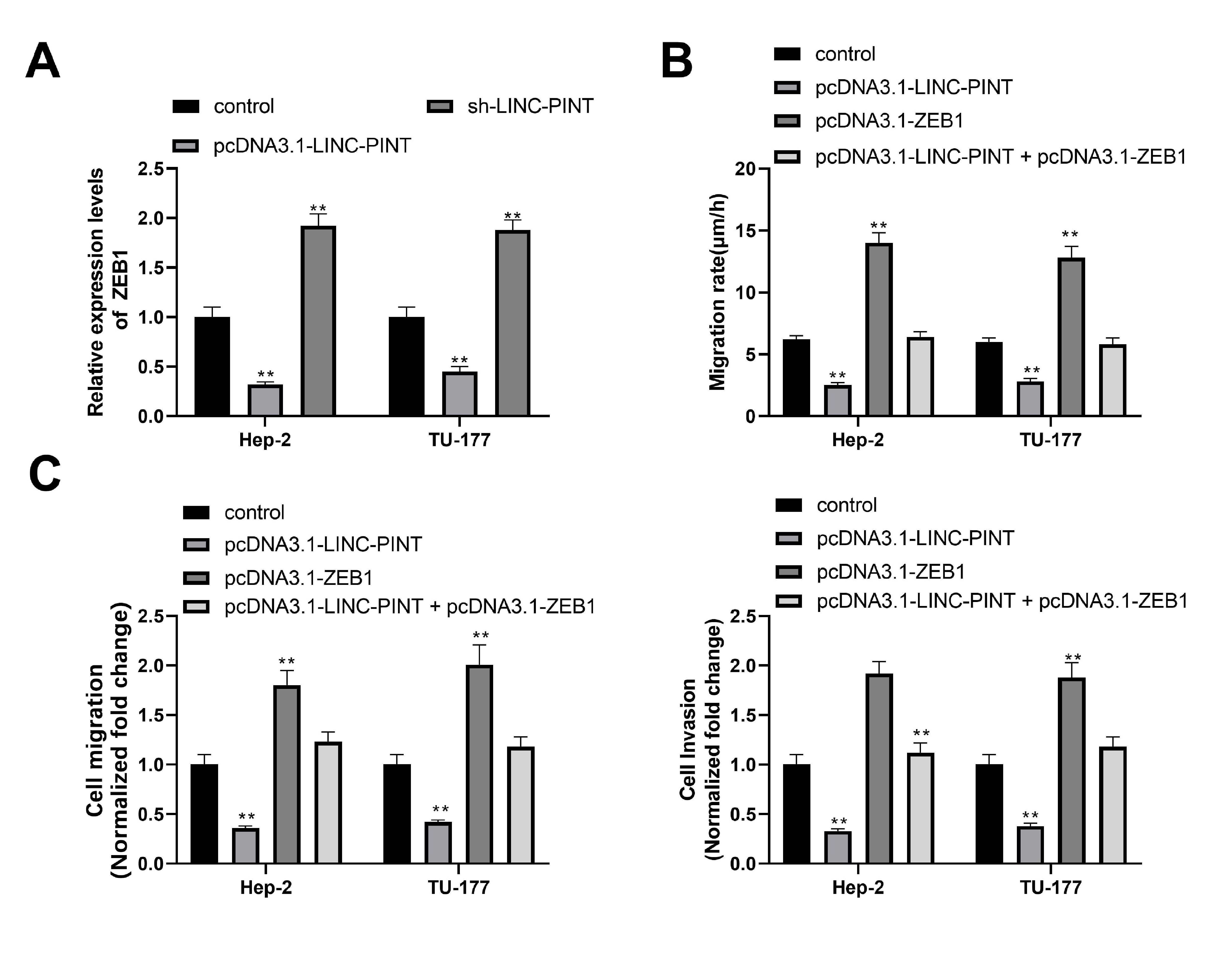

Supplement: Supplementary file 3 [file Image3.TIF]

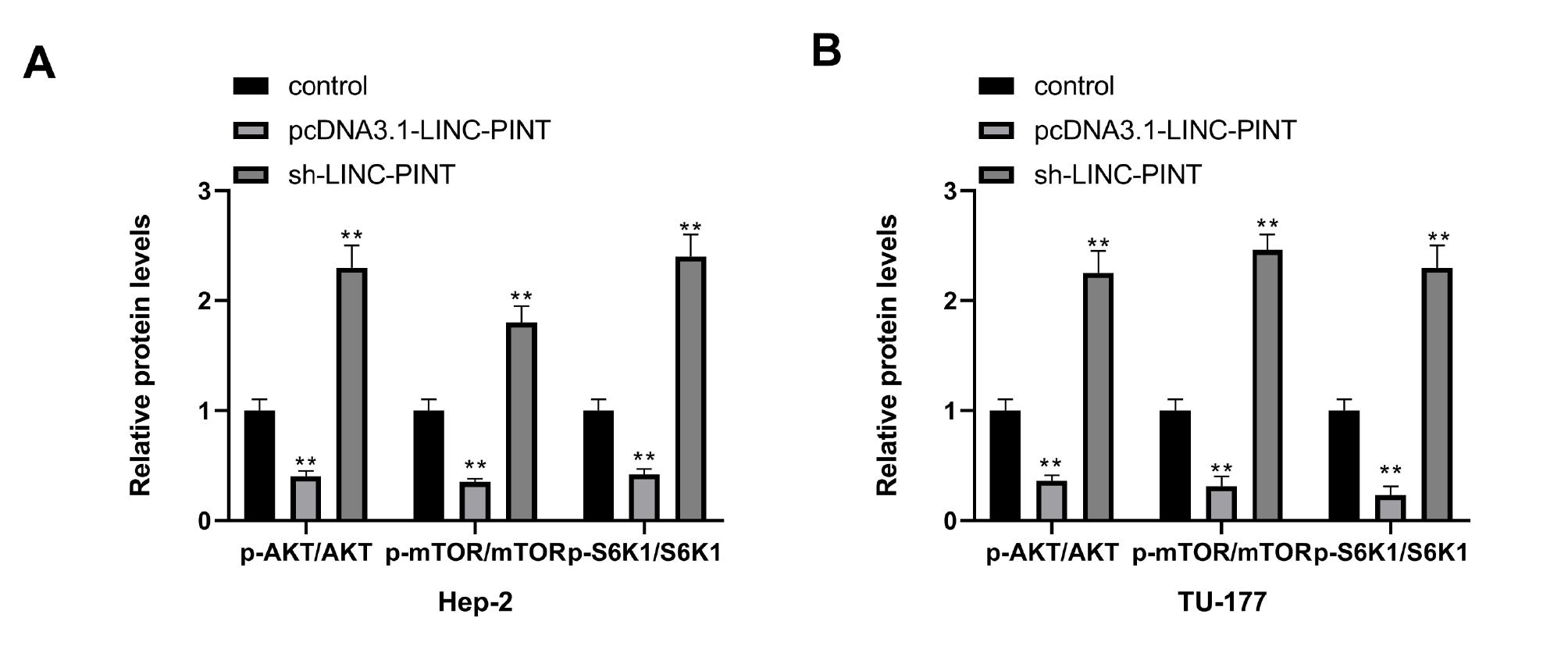

Supplement: Supplementary file 4 [file Image4.TIF]
